# Effectively suppressed angiogenesis-mediated retinoblastoma growth using celastrol nanomicelles

**DOI:** 10.1080/10717544.2020.1730522

**Published:** 2020-02-24

**Authors:** Zhanrong Li, Zhihua Guo, Dandan Chu, Huayang Feng, Junjie Zhang, Lei Zhu, Jingguo Li

**Affiliations:** Henan Eye Hospital, Henan Provincial People’s Hospital, Zhengzhou University People’s Hospital, Zhengzhou, P. R. China

**Keywords:** Celastrol, nanomicelles, retinoblastoma, SO-Rb 50 cells, angiogenesis

## Abstract

Celastrol, a Chinese herbal medicine, has already shown an inhibition effect on retinoblastoma growth activity in our previous research, but its mechanism is not well understood. Angiogenesis is a main driving force in many tumors. Here, we studied whether celastrol could inhibit angiogenesis-mediated retinoblastoma growth, if so, through what mechanism. In this work, we developed celastrol-loaded polymeric nanomicelles to improve the poor water solubility of celastrol. When given an intraperitoneal injection to mice bearing human retinoblastoma xenografts, celastrol nanomicelles (CNMs, 27.2 mg/kg/2 days) significantly reduced the weight and the volume of tumors and decreased tumor angiogenesis. We found that CNMs suppressed hypoxia-induced proliferation, migration, and invasion by human umbilical vascular endothelial cells (EA.hy 926) in a dose-dependent manner. Furthermore, CNMs inhibited SO-Rb 50 cells-induced sprouting of the vessels and vascular formation in chick embryo chorioallantoic membrane assay *in vitro*. To understand the molecular mechanism of these activities, we assessed the signaling pathways in CoCl_2_ treated EA.hy 926. CNMs inhibited the hypoxia-induced HIF-1α and VEGF. In conclusion, our results reveal that CNMs target the HIF-1α/VEGF pathway, which may be an important reason for the suppression of retinoblastoma growth and angiogenesis.

## Introduction

Retinoblastoma is the most frequently occurring intraocular malignancy of infancy and childhood with an incidence of 1 in 15,000 to 20,000 live births (Kim et al., 2019). At present, the main treatment approaches for retinoblastoma are enucleation, intravenous chemotherapy (Dimaras et al., 2015), intra-arterial chemotherapy (Abramson et al., 2010) focal treatments including laser photocoagulation, brachytherapy, transpupillary thermotherapy (Shields et al., 2014), which is based on international retinoblastoma classification and disease stage (Abramson et al., 2015). A previous study has reported that retinoblastoma tumor samples have a large amount of heterogeneous vasculature containing neovascularization and pericyte-committed mature vasculature measured by immunohistochemical analysis (Jockovich et al., 2008). Tumor angiogenesis is required to deliver nutrients and oxygen to growing tumors. It plays a key prominent in maintaining tumor growth and enabling tumor metastasis. And therefore, the development of a novel strategy that targets tumor angiogenesis has become a promising approach for improving the clinical outcome of the tumor. Recent studies have reported that tumor growth and metastasis in the lung cancer (Li et al., 2015), breast cancer (Li et al., 2016), prostate cancer, and cervical cancer could be inhibited by suppressing tumor angiogenesis. Nevertheless, the research of intervention on retinoblastoma neovascularization was rarely reported.

Over the past few decades, polymeric nanomicelles have been used as delivery vehicles for the solubilization and controlled delivery of water-insoluble drugs. Drug-loaded polymeric micelles offer favorable biological properties, such as biocompatibility, biodegradability, and controlled drug release. Recently, polymeric micelles have been used for ocular drug delivery to improve the drug release (Li et al., 2015).

Our previous study showed that celastrol nanomicelles (CNMs) inhibited the growth of retinoblastoma in mouse xenograft model through inducing the apoptosis of human retinoblastoma SO-Rb 50 cells (Li et al., 2012). We also found that CNMs suppressed effectively the corneal neovascularization (Li et al., 2012; Li et al., 2016). Whether celastrol could modulate retinoblastoma angiogenesis has not been validated yet. Celastrol, a pentacyclic triterpene extracted from Thunder of God Vine, is a potent anti-inflammatory, anti-angiogenesis, anticancer and antiobesity agent (Feng et al., 2019). Nevertheless, the further clinical application of celastrol is restricted by its poor water solubility. In the previous study, we developed celastrol-loaded nanomicelles which could significantly improve the apparent solubility of celastrol (Li et al., 2012). Therefore, in this study, we investigated the effect of CNMs on retinoblastoma angiogenesis.

Hypoxia is one of the characteristic features of the tumor microenvironment. Intratumoral hypoxia induces the expression of the vascular endothelial growth factor (VEGF), which is the well-known angiogenesis inducer, and the upregulations of VEGF under hypoxic conditions could increase microvascular density as well as permeability and even promote intravasation and extravasation. Furthermore, hypoxia selects subpopulation of the cancer cells with an invasive and metastatic phenotype that have the abilities to escape from the primary tumors. In the study of Sato et al., they mimicked hypoxia by cobalt chloride (CoCl_2_) and evaluated the effect of a redox-silent analog of tocotrienol on angiogenesis in human malignant mesothelioma (Sato et al., 2014). Bi et al. reported that the effect of gamma-tocotrienol on angiogenesis in human gastric adenocarcinoma induced by CoCl_2_ (as a hypoxia mimic) (Bi et al. 2010). In this study, we evaluated the efficacy of celastrol on retinoblastoma angiogenesis in vivo. *In vitro*, CoCl_2_ treatment was used to simulate the hypoxic environment of the tumor, to further determine its effect on hypoxia-induced vascular endothelial cells.

## Material and methods

### Preparation of CNMs

**Figure 1.**
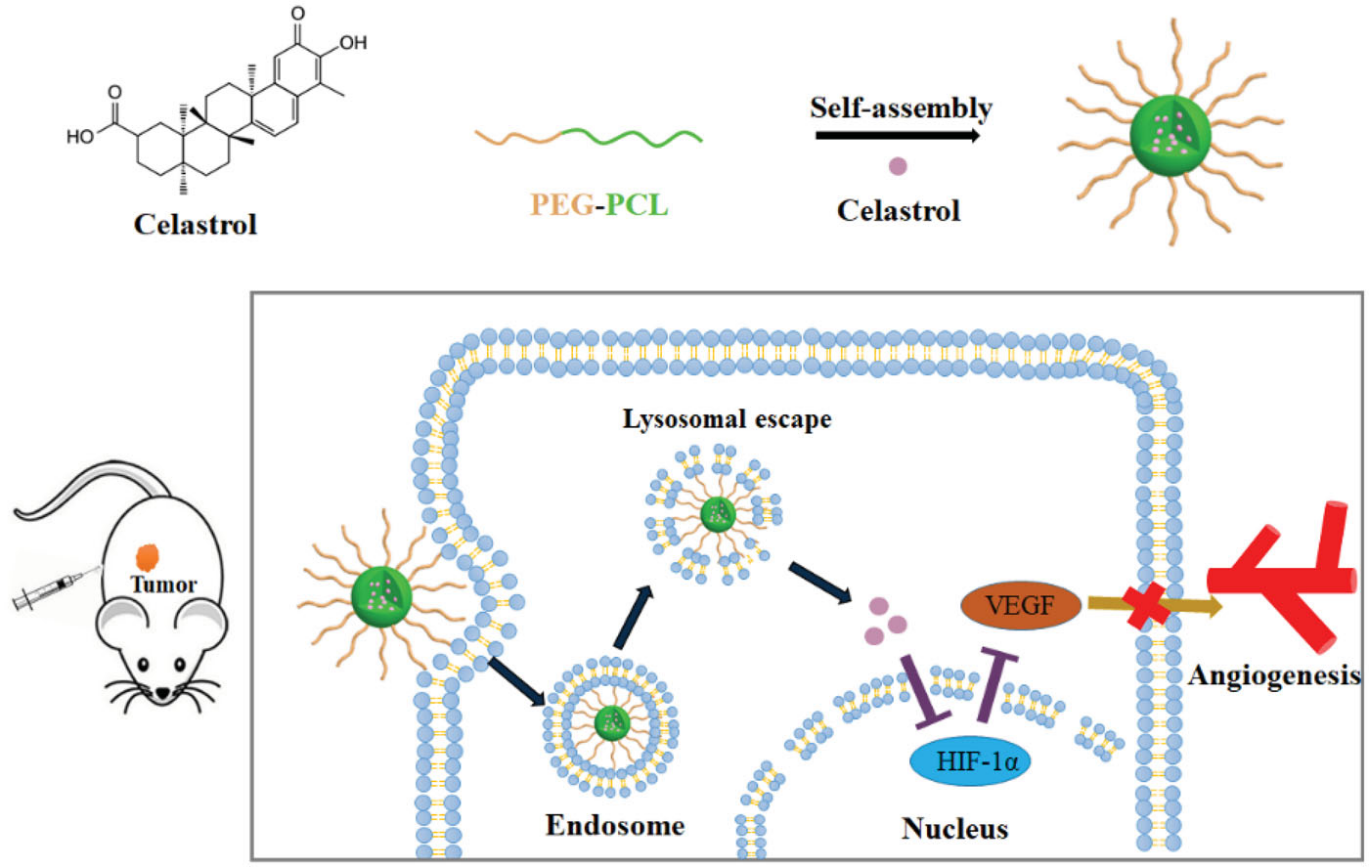
Schematic illustration of the preparation of celastrol nanomicelles for celastrol delivery, resulting in suppressing retinoblastoma growth and angiogenesis by the HIF-1α/VEGF pathway.

Celastrol (Figure 1) was purchased from the Shanghai Institute of Materia Medica, Chinese Academy of Sciences (Shanghai, China). The preparation of celastrol loaded polymer micelles was performed as previously described (Li et al., 2012). Briefly, celastrol-loaded nanomicelles were prepared by dissolving PEG-b-PCL (2000:1000, Mw/Mn = 1.18, JCS Biopolytech, Toronto, Canada; Figure 1) (10 mg) and celastrol (2 mg) in chloroform (2 mL), and then adding the solution dropwise to double-distilled water (20 mL) under ultrasonic agitation using a Sonic Dismembrator (Type 60, Fisher Scientific, Pittsburgh, PA). The organic solvent was then removed through vacuum distillation using a rotary evaporator to allow micelle formation. The samples were further concentrated and washed 3 times to remove free celastrol dissolved in the micelle solution through a 0.22 µm polyether sulfone filter. The schematic illustration of CNMs formation is shown in Figure 1.

### Cell line and cell culture

The human umbilical vein endothelial cells (HUVECs) EA.hy 926 (American Type Culture Collection [ATCC], Manassas, VA, USA) were cultured in Dulbecco’s modified Eagle’s medium (DMEM) containing 10% fetal bovine serum (FBS, Gibco, Gaithersburg, MD), 100 U/mL penicillin, 100 µg/mL streptomycin, as well as 4.5 g/L glucose at 37 °C in 5% carbon dioxide atmosphere. SO-Rb 50 (human retinoblastoma cell line) cells were presented by Professor Yongping Li of Sun Yat-sen University, and were cultured in RPMI-1640 medium containing 10% FBS (Gibco, Gaithersburg, MD), 100 U/mL penicillin, and 100 µg/mL streptomycin under the same conditions at 37 °C in 5% carbon dioxide.

### Cell migration assay

HUVECs migration assay was performed as described previously (Feng et al., 2019). Briefly, EA.hy 926 cells were allowed to grow to 90% confluence in gelatin-coated 6-well plates and then incubated with DMEM containing 0.5% FBS for 6 h to inactivate cell proliferation. EA.hy 926 cells were scratched with pipette tips in medium and washed with phosphate-buffered saline (PBS). DMEM containing 0.5% FBS was added into the wells with different concentrations CNMs (0, 1, 2, and 5 µg/mL) under hypoxia. After 16 hours of incubation, images of the cells were taken using an inverted microscope (Olympus). The migrated cells were quantified manually, and the percentage inhibition was expressed using untreated wells as 100%.

### Cell invasion assay

The chemotactic motility of EA.hy 926 cells was determined using invasion assay with 24-well 8-µm pore size transwell chambers (Corning Incorporated). In brief, EA.hy 926 cells (4 × 10^4^/well) were seeded in the top chambers, which were coated with 0.1% gelatin. The bottom chambers were filled with DMEM containing 0.5% FBS supplemented with different concentrations of CNMs (0, 13.6, and 27.2 µg/mL). EA.hy 926 cells were incubated for 8 hours at 37 °C, 5% CO_2_, cells on the top chambers (non-invaded) were removed using cotton swabs, and cells on the bottom chambers (invaded) were fixed with cold 4% paraformaldehyde and stained with 0.05% crystal violet. Images were obtained using an inverted microscope (Olympus). Crystal violet-positive invaded cells were quantified by manual counting. Percentage inhibition of invading cells was quantified and expressed based on untreated control wells. Three independent experiments were performed.

### HUVECs proliferation assay

The inhibitory effects of CNMs on HUVECs proliferation were measured by using the tetrazolium monosodium salt (WST-8) reagent 2-[2-methoxy-4- nitrophenyl]-3-[4-nitrophenyl]-5-[2,4-disulfophenyl]-2H (Nacalai Tesque, Kyoto, Japan), following the manufacturer’s instruction. EA.hy 926 cells were seeded in 96-well cell culture plates at a density of 4 × 10^3^ cells/well in triplicate in fresh medium incubate for 24 hours. After that, EA.hy 926 cells were treated with medium containing CoCl_2_ (100 µmol/L) for 24 hours. EA.hy 926 cells were respectively treated with medium containingdifferent concentrations of CNMs 0, 1, 2, 4, 8, and 16 µg/mL; celastrol loading content: 7.36%) and then incubated for an extra 72 hours. WST solution was applied at 10 µL/well, and the cells were incubated for 4 hours at 37 °C. The absorbance values of all wells were then measured at 450 nm.

### Western blot analysis

EA.hy 926 cells were treated with CNMs (27.2 µg/mL) and an equal amount of PEG-b-PCL nanomicelles without celastrol in hypoxic conditions for 24 hours to determining vascular endothelial growth factor A (VEGF-A) (Abcam, USA) and hypoxia-inducible factors-1α (HIF-1α) (Abcam, USA) protein expression. Hypoxic conditions were created by CoCl_2_ (100 µmol/L). The cells were collected, lysed, and the proteins were extracted according to the manufacturer’s instructions. The concentrations of protein were measured by the BCA method. The same amount of each sample was loaded on sodium dodecyl sulfate gels and transferred onto PVDF membranes. Membranes were blocked with 5% nonfat milk at room temperature for 2 hours, and incubated with diluted primary antibodies VEGF-A, HIF-1α, glyceraldehyde 3-phosphate dehydrogenase (GAPDH) (Abcam, USA) (1:1000) specific for the target proteins overnight at 4 °C. After washing, the membranes were incubated with HRP-labelled secondary anti-rabbit or anti-mouse antibodies for 1 hour at room temperature and were then visualized by enhanced chemiluminescent (ECL). GAPDH was used as loading controls.

### Chick embryo chorioallantoic membrane assay

Chick embryo chorioallantoic membrane (CAM) assays were performed as described previously (Ribatti et al., 2006). Fertilized chicken eggs were incubated at 37 °C and 60% humidity in an egg incubator for 48 hours. The neovascularization of CAM was induced by retinoblastoma SO-Rb 50 cells, then treated with various concentrations of CNMs (0, 3.4, 6.8, and 13.6 µg/mL) continuously for 48 hours, then evaluated and recorded by using stereomicroscope equipped with a camera and image analysis system (Olympus). The antiangiogenic response was determined by analyzing the branching of blood vessels.

### Xenograft mouse model

All the mice care and experimental protocols in this study were following the Association for Research in Vision and Ophthalmology Statement and the Ethical Committee of Experimental Animal Care of Henan Eye Institute. Retinoblastoma mouse models were constructed as previously described (Li et al., 2012). 5- to 6-week-old female NOD-SCID mice were randomly divided into two groups of five each. SO-Rb 50 cells were subcutaneously injected into the right flanks of mice (5 × 10^7^ cells/mouse). After tumors grew to a mean tumor volume of 100–200 mm^3^, following the formula: volume = length × width × 0.5 (Dimaras et al., 2015). The CNMs group received intraperitoneal injections of CNMs (27.2 mg/kg/2 days); the control group was injected with the equal-dose of PEG-b-PCL nanomicelles without celastrol through intraperitoneal injection. The size of the tumor was determined by Vernier calipers every 4 days. Based on the animal protocols, the mice were euthanized when the tumor size was reached >1000 mm^3^.

### Histological analysis

Tumor tissue was removed fixed with 10% formaldehyde and then embedded with paraffin. To identify the changes of blood vessels, the tissue was cut into 4-µm-thick sections and stained with hematoxylin and eosin. Immunohistochemistry for CD31 (Cell signaling Technology) was carried out on deparaffinized sections to identify vascular endothelial cells. Thereafter, morphology and images of the tissue were assessed by light microscopy.

### Statistical analysis

The data were presented as mean ± standard deviation, and statistical analysis was performed using IBM SPSS software for Windows (v 16.0; SPSS Inc, Chicago, IL, USA). The significance of differences between the control group and the CNMs treated group was determined by the independent-sample student’s *t*-test. *p* values < 0.05 were considered statistically significant.

## Results

### Characterization of nano-micelles and celastrol loading content

The characterization of nano-micelles and celastrol loading content were determined in our previous study (Li et al., 2015). The size and zeta potential of the celastrol-loaded nano-micelles were measured via dynamic light scattering, and the results are indicated that the celastrol-loaded nano-micelles had a relatively small average hydrodynamic diameter of 48 nm. The morphology of micelles illustrated their uniform spherical shape. The celastrol loading content of CNMs was 7.36%, and the study of *in vitro* celastrol release showed controlled release behavior.

### CNMs suppressed HUVECs migration and invasion

**Figure 2.**
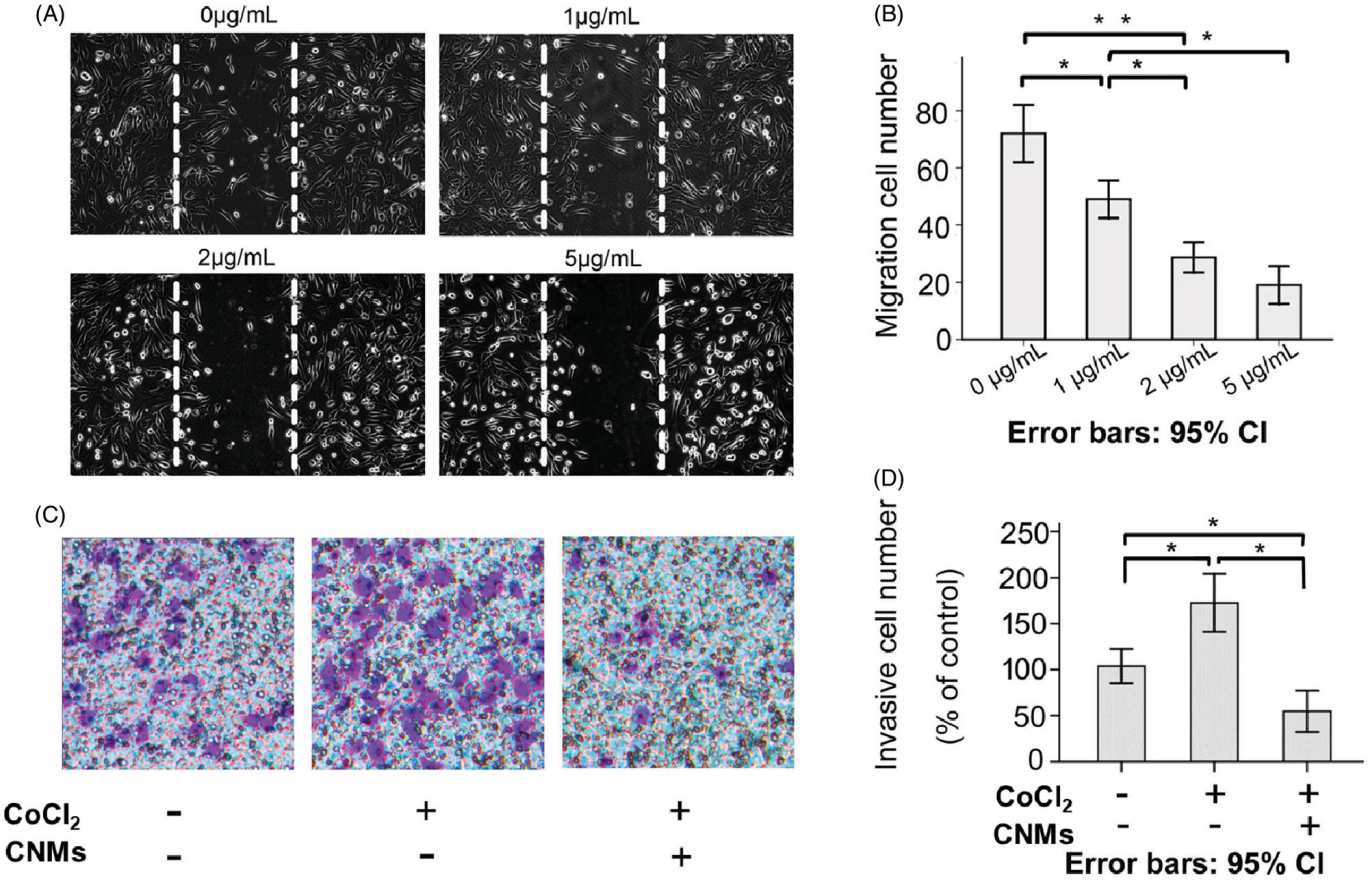
CNMs inhibit hypoxia-inducible migration and invasion of endothelial cells. (A) CNMs inhibited vascular endothelial cells migration. Representative images of CNMs inhibiting EA.hy 926 cells migration in the scratching assay (magnification: × 200). (B) The quantitative analysis of migration (*p < 0.05, **p < 0.01). (C) CNMs inhibited endothelial cells invasion. Representative images of CNMs inhibiting EA.hy 926 cells invasion. cell migration in the Transwell chamber assay (magnification: × 200). (D) The quantitative analysis of invasion (*p < 0.05).

Endothelial cell migration is an essential step during tumor angiogenesis. To assess the anti-angiogenic properties of CNMs on hypoxia-induced HUVECs migration, we examined the HUVECs migration using the wound-healing migration assay. The results showed that migration cell number was 72.0 ± 4.0 in the blank micelles group and 49.0 ± 2.6, 28.7 ± 2.1 and 19.0 ± 2.6 at different concentrations of CNMs (1, 2 and 5 µg/mL, respectively, *p < 0.05, **p < 0.01; Figure 2(A,B)) under hypoxia. We concluded that wound healing stimulated by hypoxia was inhibited in a concentration-dependent manner after treated with CNMs.

Hypoxia was a common condition of the tumor microenvironment, which involved in tumor cell invasion and metastasis. We examined whether CNMs could suppress the invasion ability of EA.hy 926 cells by the Matrigel-coated chamber transwell assay. The Transwell results showed that invasive cell number was 103.81 ± 7.50 in the blank micelles group (0 µg/mL), 172.56 ± 12.63 in the hypoxia-induced group and 54.76 ± 8.98 at the concentrations of CNMs-treated hypoxia-induced group, (*p < 0.05, Figure 2(C,D) respectively. The invasive cell numbers were statistically significantly different between the blank micelles group and the hypoxia-induced group. Meanwhile, an obvious reduction was observed in the CNMs-treated hypoxia-induced group compared with the hypoxia-induced group and blank micelles group, indicating that the invasion capacity of the EA.hy 926 cells after the treated with CNMs was significantly decreased.

### CNMs inhibit the proliferation of HUVECs in vitro

**Figure 3.**
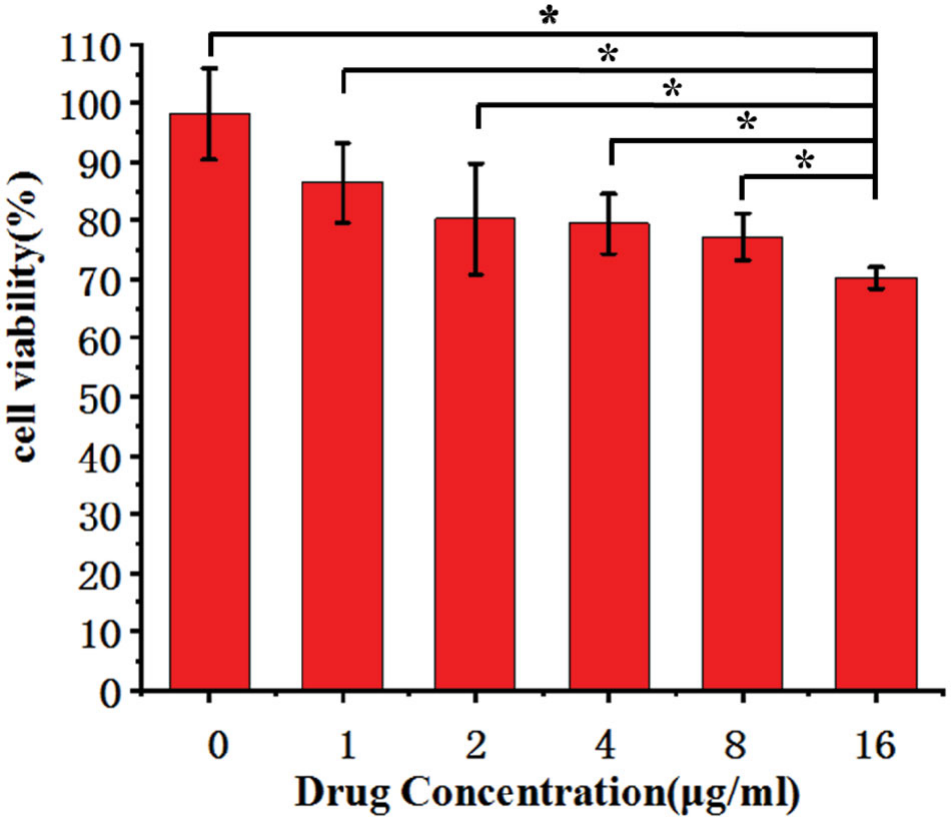
Celastrol nanomicelles inhibit the growth of human umbilical vein endothelial cells EA.hy 926 treated CoCl_2_ (100 µmol/L) for 72 hours. (*p < 0.05).

The cells proliferation inhibition effects of CNMs on EA.hy 926 cells were detected using WST assays. Under the hypoxic condition, at various concentration of CNMs (0, 1, 2, 4, 8, and 16 µg/mL), the viability of EA.hy 926 cells was decreased. (Figure 3).

### CNMs inhibit the HIF-1α and VEGF-A pathways in hypoxia-induced HUVECs

**Figure 4.**
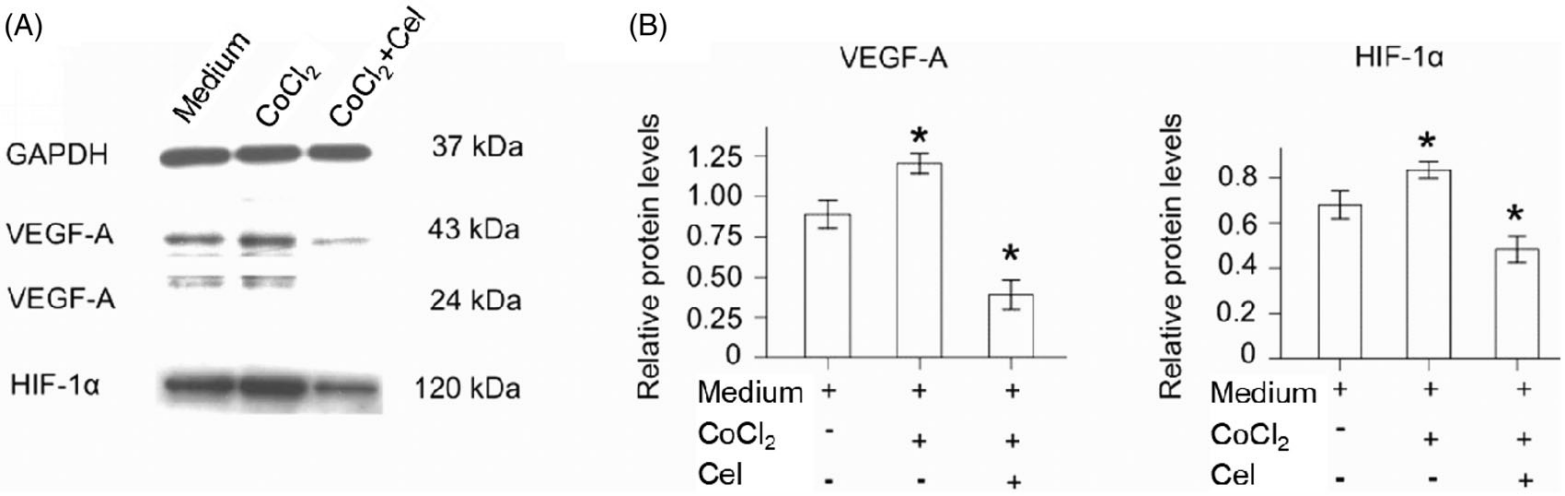
CNMs suppress hypoxia-inducible VEGF-A and HIF-1α protein expression in EA.hy 926.7 cells treated with CoCl_2_. EA.hy 926.7 cells were incubated in the presence or absence of 27.2 µg/mL CNMs for 24 hours under normal or hypoxic (CoCl_2_) conditions. VEGF-A and HIF-1α protein levels were detected by western blotting. (A) shows one representative western blot of three independent experiments. (B) show the relative protein levels and the statistical analysis results (mean values ± SD). cytosolic and nuclear fractions under normal conditions without CNMs were set as 1, *p < 0.05.

Western blot analysis showed that HIF-1α, VEGF-A expression was increased in hypoxia-induced HUVECs compared with normal HUVECs. In addition, expression of HIF-1α, VEGF-A was decreased in CNM-treated EA.hy 926 cells compared with the other two groups of EA.hy 926 cells (Figure 4, *p < 0.05). HIF-1α protein expression was increased by 1.37 ± 0.04-fold (*n* = 3) under hypoxia and was reduced by 0.43 ± 0.03-fold (*n* = 3) in the presence of 27.2 µg/mL CNMs compared with the control. Furthermore, VEGF-A protein expression was increased by 1.17 ± 0.06-fold and 1.57 ± 0.06-fold (*n* = 3), respectively, under hypoxia and was decreased by 0.17 ± 0.02-fold and 0.27 ± 0.06-fold (*n* = 3) after CNMs treatment (*p < 0.05). The high level of HIF-1α in tumors could promote erythropoiesis and angiogenesis via increasing oxygen availability. Transcriptional regulation of VEGF is critically dependent on HIF-1a. Furthermore, VEGF-A was one of the most potent angiogenic factors (Kuchta et al., 2017). Therefore, HIF-1α and VEGF-A have been determined as critical mediators in the angiogenesis process. In the present study, the results indicated that treatment with CNMs obviously decreased the CoCl_2_-induced over-expression of HIF-1α and VEGF-A in EA.hy 926 cells, which applied its function by the down-regulating HIF-1α and VEGF-A expression. It could be concluded that CNMs inhibited tumor angiogenesis through the blocking of the VEGF-A pathway.

### Angiogenesis of CNMs in chick chorioallantoic membrane assays

**Figure 5.**
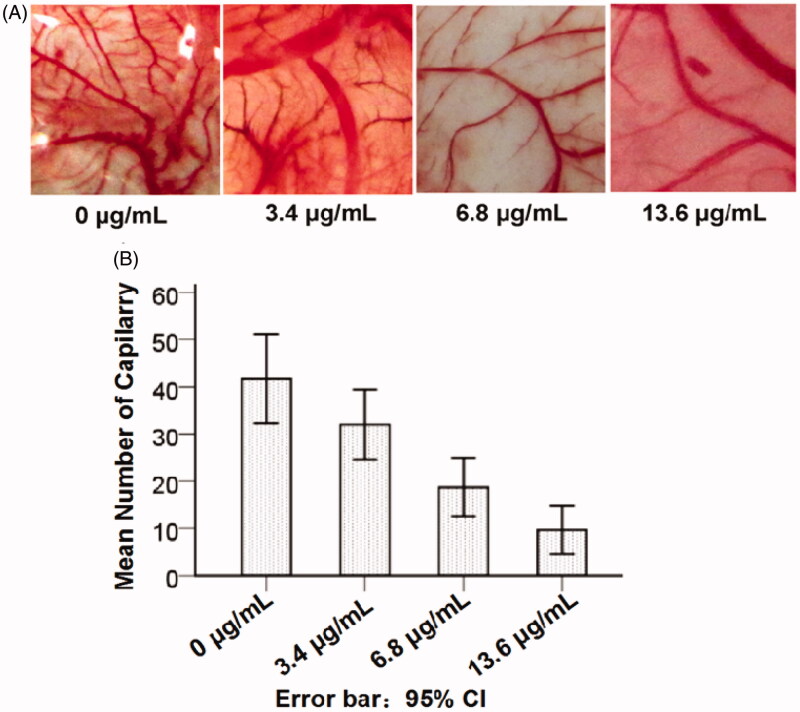
CNMs inhibited angiogenesis in the chick chorioallantoic membrane (CAM). The neovascularization of CAM was induced by retinoblastoma SO-Rb 50 cells *in vitro*, treated with various concentrations of CNMs (0, 3.4, 6.8, and 13.6 µg/mL) continuously for 48 hours, then recorded by using stereomicroscope (Olympus). Representative photographs of CAM assays.

Chick chorioallantoic membrane (CAM) assays were the most widely used model *in vitro* to study the angiogenic process and effects of anti-angiogenic agents. Therefore, CAM assays were used to investigate the effects of CNMs *in vitro*. Compared with the blank micelles group, neovascularization of CAM treated with various concentrations of CNMs (3.4, 6.8 and 13.6 µg/mL) was dramatically decreased in a concentration-dependent manner (Figure 5), suggesting that CNMs could suppress angiogenesis *in vitro*.

### CNMs suppress tumor growth in a xenograft mouse model

**Figure 6.**
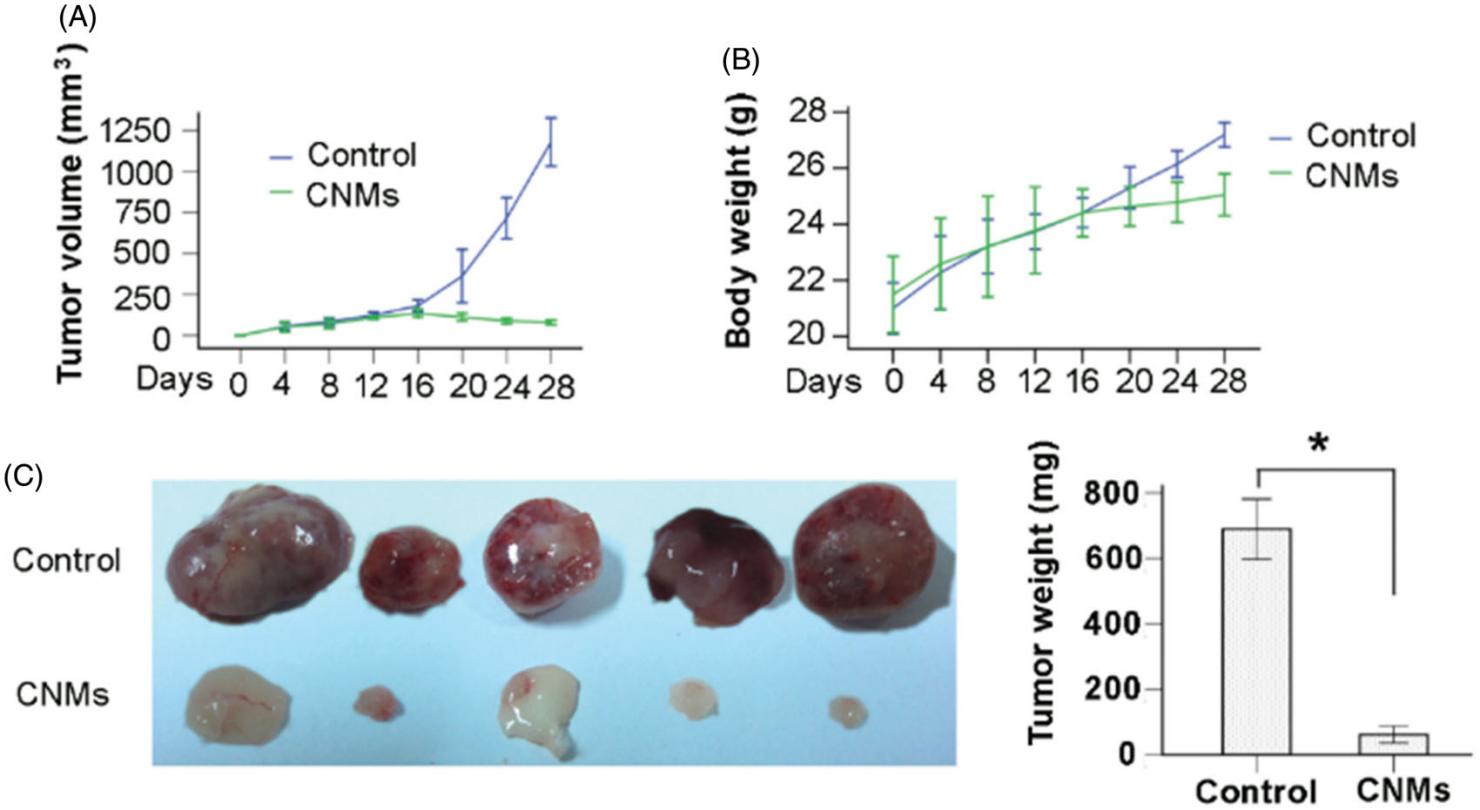
CNMs suppress retinoblastoma growth in a xenograft mouse model. SO-Rb 50 cells were subcutaneously injected into 6-week-old NOD SCID mice (5 × 10^7^ per mouse). The mice were administrated CNMs or blank micelles (27.2 mg/kg/2 days) by intraperitoneal injections after solid tumors grew to 100 mm^3^. (A) CNMs inhibit tumor growth as measured by tumor volume. (B) As demonstrated by the change in body weight, CNMs had little toxicity in mice at the doses tested. (C) Solid tumors in mice treated with CNMs were significantly smaller than those in control mice. *p < 0.01 versus control. Error bars: 95% CI.

To investigate the effect of Celastrol on tumor growth in vivo, we applied CNMs in a retinoblastoma tumor (SO-Rb 50 cells) xenograft model. We found that administration of 27.2 mg/kg CNMs every other day for 16 days substantially suppressed tumor volume and reduced tumor weight (Figure 6). The average tumor volume in the control mice increased from 180.87 ± 29.34 to 1178.90 ± 118.83 mm^3^ after 16 days, whereas the average tumor volume in the CNMs-treated mice decreased from 135.19 ± 19.10 to 79.40 ± 11.35 mm^3^ (Figure 6(A)). Additionally, the average tumor weight in the control group was 689.60 ± 73.95 mg, whereas the average tumor weight in CNMs-treated group was only 61.40 ± 20.82 mg (Figure 6(B)), suggesting a significant inhibition in tumor growth. However, the equal dose of CNMs had no obvious effect on the body weight of mice compared to the control group (Figure 6(C)).

### Histological examination

**Figure 7.**
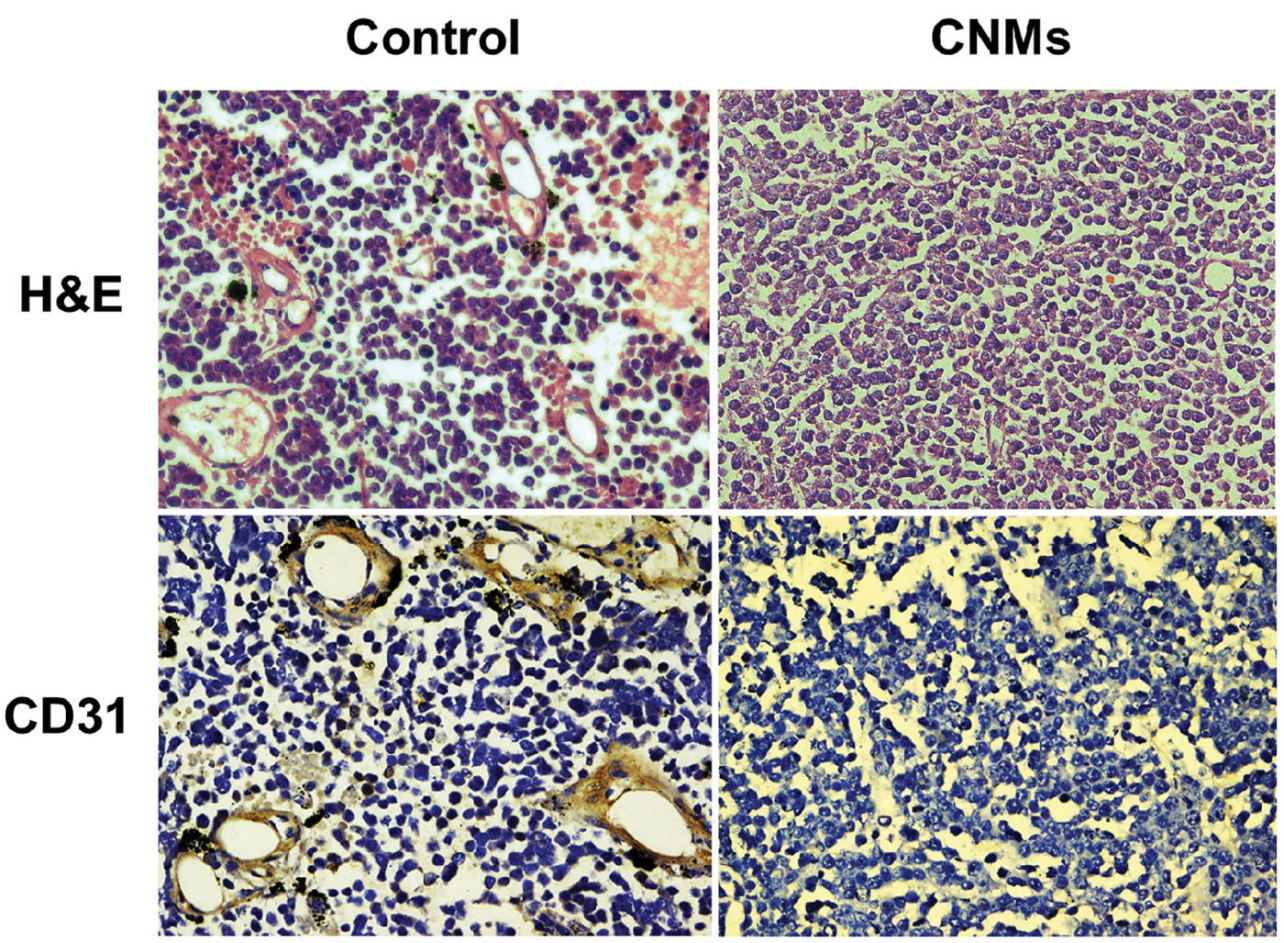
CNMs inhibited neovascularization in retinoblastoma in mice as measured by H&E staining analysis and CD31 staining.

In retinoblastoma tissue sections, we used hematoxylin and eosin (H&E) staining to quantify the amount of functional vasculature. The vascular lumen was significantly reduced in CNMs-treated group when compared with the control group (Figure 7). CD31 was a microvascular marker expressed in vascular tumors. Immunohistochemistry staining of CNMs-treated retinoblastoma tissue sections with CD31 showed a decreased tumor vascularization compared with the control group (Figure 7). The results indicated that CNMs significantly reduced tumor vascular density and inhibited angiogenesis in retinoblastoma.

## Discussion

Celastrol, a component of traditional Chinese medicine, has been demonstrated that exhibit potent anti-tumor (Nanjundaiah et al., 2012), anti-inflammation, anti-angiogenesis activity (Hanahan & Weinberg, 2011). Our previous study indicated that celastrol nanoparticles can significantly suppress the growth of retinoblastoma. With further research, we found that celastrol nanoparticles inhibit retinoblastoma growth through inducing the apoptosis of human retinoblastoma SO-Rb 50 cells (Li et al., 2015). Meanwhile, immunohistochemical analysis of the retinoblastoma pathological tissue sections showed that a large amount of neovascularization form in retinoblastoma tissue sections of the control group, but significantly reduced in CNMs treated group. In the present study, we concentrated on studying the effect of CNMs on the neovascularization of retinoblastoma, and further investigated the mechanism of CNMs effects against retinoblastoma.

Angiogenesis is essential for many pathologic processes, especially for the growth and metastasis of the tumor. Previous research showed that angiogenesis is induced astonishing early in the multistage development of invasive cancers in humans and animal models (Nyberg et al., 2005). Tumor angiogenesis is generally characterized by excessive neovascularization and provide nutrition and oxygen needed by tumor cells which can promote the growth, metastasis, and invasion of tumors (Li et al., 2017). Recently, niclosamide, tigecycline, and quercetin have been reported to be potential candidates for treating retinoblastoma via suppressing the proliferation of retinoblastoma cells and aspects of retinoblastoma angiogenesis (Huang et al., 2011). In the researches of celastrol on the tumor, celastrol inhibits tumor growth through multiple mechanisms. For instance, we have demonstrated that celastrol inhibits retinoblastoma growth by inducing apoptosis in human retinoblastoma SO-Rb 50 cells (Li et al., 2015). Besides, the other research showed celastrol can suppress the growth of prostate cancer through inhibition of tumor angiogenesis (Gilkes et al., 2014). In the present study, our results have shown that CNMs can significantly suppress retinoblastoma tumor growth and neovascularization in vivo. In the CAM assays, our further investigation indicated that CNMs can inhibit neovascularization of CAM induced by retinoblastoma SO-Rb 50 cells *in vitro*. The results showed that CNMs possess strong anti-angiogenesis effects. In the previous study, we found that celastrol significantly inhibit suture-induced corneal neovascularization by suppressing macrophage infiltration (Li et al., 2012). Besides, CNMs potently suppressed macrophage-induced corneal neovascularization in rats through the inhibition of cytokine secretion in macrophage (Li et al., 2012). Therefore, we speculated that CNMs could suppress the growth of retinoblastoma via inhibiting the formation of neovascularization.

New blood vessel formation in the tumor is required to provide sufficient oxygen, nutrients, and metabolites for the rapid proliferation of tumor cells. As the sustained expansion of tumor mass, diffusion distances from the existing vascular supply increases resulting in the formation of hypoxia-ischemia microenvironment in the tumor tissues (Gilkes et al., 2014). Hence, hypoxia is an obvious feature for the tumor microenvironment. The hypoxia-inducible factors-α (HIF-α) (including HIF-1α and HIF-2α) regulate the adaptation of tumor- and metastasis-initiating cells to the oxygen and nutrient deprivation in tumor progression under normoxic and hypoxic conditions (Schönenberger and Kovacs, 2015; Li et al., 2019). The activity of HIF-α transcription factors is regulated by prolyl hydroxylase domain-containing proteins. The oxygen-dependent degradation (ODD) domain was a 200 amino acid sequence of HIF-α proteins. The hydroxylation of proline residues in the ODD domain was performed by proline hydroxylases under the normoxia conditions. Following, the von Hippel-Lindau protein (pVHL), a tumor suppressor protein, mediates the ubiquitination and degradation of HIF-α via interacting with the ODD domain in the oxygen conditions. The cobalt can replace iron at this site of the proline hydroxylases, which inhibit the activity of the HIF-specific proline hydroxylases. Meanwhile, cobalt prevented the interaction between hydroxylated HIF-α and pVHL, which suppressed subsequent ubiquitination as well as degradation of HIF-α and stabilized the expression of HIF-α. The major regulator of the hypoxic response is the transcription factor hypoxia-inducible factor-1α (HIF-1α), and a previous study has shown that HIF-1α accumulates rapidly during CoCl_2_-stimulated hypoxia to induce a hypoxic-like response in tumor cells (Harrison et al., 2018). Meanwhile, Sinnberg et al. have reported that CoCl_2_ was used as an inducer for HIF-1α to evaluate the cytotoxic effects of ascorbate on cancer cells (Sinnberg et al., 2014). Hence, CoCl_2_ treatment is frequently used to simulate the hypoxic environment of the tumor.

The tumor microenvironment is a relatively hypoxic environment. Intratumoral hypoxia upregulates the expression of VEGF, which can induce the angiogenesis in tumor (Li et al., 2016). Sato A, et al. also found that CoCl_2_-induced hypoxia treatment results in increased the secretion of VEGF in tumor cells (Feng et al., 2019). Once angiogenesis has been activated, tumors exhibit various forms of neovascularization which play a key role in promoting tumor growth, metastasis and invasion. And poor prognosis of retinoblastoma is closely connected with the invasion and migration, which account for the major reasons for the incidence and mortality rate (Teixo et al., 2015). Therefore, this study focused on investigating the effect of CNMs on angiogenesis-mediated retinoblastoma growth through evaluating the effect of CNMs on the vascular endothelial cell in hypoxic tumor microenvironment induced by CoCl_2_ *in vitro*. Huang and colleagues have demonstrated that celastrol could suppress hypoxia-mediated angiogenesis and metastasis by means of blocking the migration and tube formation of vascular endothelial cells as well as the invasiveness of cancer cells (Li et al., 2017). Moreover, Ni H and colleagues found that celastrol can downregulate the secretion of VEGF and block the angiogenesis *in vitro* by inhibiting EA.hy 926 cells motility and invasion (Ni et al., 2014).

In our current work, the EA.hy 926 cells were treated with CoCl_2_ to induce the hypoxia, an *in vitro* WST assay demonstrated that CNMs inhibited the proliferation of EA.hy 926 cells under hypoxia in a dose-dependent manner. Additionally, we further found that CNMs can effectively inhibit the migration and invasion of EA.hy 926 cells in hypoxic condition. These results indicate that the inhibition of CNMs on the vascular endothelial cell in hypoxic tumor microenvironment induced by CoCl_2_ *in vitro*. The overexpression of HIF-1α is associated with increased mortality in patients with a variety of tumors; this association is primarily based on the HIF-1-mediated regulation of genes that play pivotal roles in the central features of cancer pathogenesis such as angiogenesis, invasion, metastasis, and anti-apoptosis. The researchers have reported that celastrol showed a dose-dependent inhibition of HIF-1α levels under hypoxia induced by CoCl_2_ in human hepatoma cells. VEGF-A can regulate most of the endothelial response, such as the proliferation and migration of endothelial cells and vascular permeability. Furthermore, VEGF-A signaling also has a major role in the progression of angiogenesis-dependent diseases, especially cancer (Blanco and Gerhardt, 2013). In the present study, the western results showed that CNMs suppress the overexpression of HIF-1α and VEGF-A induced by CoCl_2_ in EA.hy 926 cells.

## Conclusion

In this study, we demonstrate that CNMs suppressed hypoxia-induced proliferation, migration, and invasion by human umbilical vascular endothelial cells in a dose-dependent manner. Furthermore, CNMs inhibited SO-Rb 50 cells-induced sprouting of the vessels and vascular formation in chick embryo chorioallantoic membrane assay *in vitro*. CNMs inhibit retinoblastoma growth in a xenograft mouse model by suppressing tumor angiogenesis which may be related to an inhibition in the HIF-1α/VEGF pathway. CNMs may represent a potential alternative treatment for retinoblastoma.
